# How do working conditions affect the turnover intention of medical social workers in China?

**DOI:** 10.1186/s12913-021-07435-8

**Published:** 2022-01-14

**Authors:** Na Li, Jin Peng, Rui Yang

**Affiliations:** 1 School of Humanities and Social Sciences, Xi’an Jiaotong University, Xianning West Road 28, Beilin District, Xi’an, 710049 Shaanxi China; 2 School of Public Policy and Administration, Xi’an Jiaotong University, Xianning West Road 28, Beilin District, Xi’an, 710049 Shaanxi China

**Keywords:** Working conditions, Turnover intention, Medical social workers, Job demands-resources model, Price-Mueller turnover model

## Abstract

**Background:**

The development of medical social work is an indispensable part of the Healthy China Strategy. However, the medical service field has the fewest social workers in all service fields in China. Creating favorable working conditions can reduce the turnover intention of social workers in the medical service field. So it is necessary to integrate the existing theoretical models to deeply analyze the multiple influencing paths of working conditions on the medical social workers’ turnover intention in the context of China.

**Methods:**

The data we used came from the China Social Work Longitudinal Survey (CSWLS) conducted in 56 cities across the country in 2019. It adopted a multi-stage random sampling method and the sample of medical social workers was selected according to their current service field and the sample size finally entering the model was 382. We tested the relationships with the Structural Equation Model (SEM) by STATA 16.0.

**Results:**

Job-related stress play the most significant role in explaining the formation mechanism of medical social workers’ turnover intention. On the one hand, job-related stress can reduce the job satisfaction of medical social workers, further increasing their turnover intention; on the other hand, job-related stress can increase job burnout of medical social workers, further reducing their job satisfaction and ultimately increasing the turnover intention. Job satisfaction plays a full mediating effect between the job burnout of medical social workers and their turnover intention. The social support and job autonomy provided by social work agencies have limited effects on decreasing the turnover intention of medical social workers.

**Conclusions:**

The two paths of job-related stress affecting turnover intention successfully integrate the Job Demands-Resources Model and the Price-Mueller Turnover Model into the same theoretical framework providing a theoretical basis for reducing the turnover intention and behavior of social workers in the medical service field, improving the management level in the medical service system and promoting the overall healthy and sustainable development of medical social work in China.

## Background

As an indispensable part of the modern medical and health care system, medical social work is mainly referred to the occupational activities that social workers use professional values and methods to help the patients and their families to prevent, mitigate and solve their emotional, psychological, and social problems caused by disease, to further improve the medical outcomes and the level of public health [1, 2]. The healthy and orderly development of medical social work is conducive to the transformation from “disease-centered” to “health-centered” of the Chinese medical and health service system [3], and it is very crucial to improve the level of health service supply in China and to ensure people’s health in an all-round and whole life cycle.

Currently, the development of medical social work in China is still in the exploratory stage. According to the China Social Work Longitudinal Survey (CSWLS) conducted in 56 cities across the country in 2019, social workers engaged in medical services only account for 5.6% of the total sample (*n* = 6776), the fewest of all service fields [4]. The insufficiency of the number of medical social workers and the lack of a clear career development path lead to difficulties to recruit and retain social workers in the medical service field in China. It will further affect the quality, consistency, and stability of medical services [5]. In terms of the working conditions of medical social workers in China, the managers and staff in the health system showing less recognition and support on medical social work, the service objects and the public having less awareness of medical social workers, and the complex working content in medical settings, these all probably increase the turnover intention of medical social workers at the individual level [2]. The relationship between working conditions and turnover intention is a key indicator to predict turnover behavior [6], since “intention precedes leaving” [7]. As a result, increasing attention has been paid to the study of the relationship between working conditions and turnover intention. The influencing paths of the working conditions on the employees’ turnover intention vary with the difference of service fields and contexts. As mentioned above, the working conditions of medical social work in China has its own particularities, and there is currently a lack of research on medical social workers’ turnover intention in a Chinese context. Therefore, it is necessary to explore the experience of professionals in this field of China.

In theory, The Job Demands-Resources Model [8] and the Price-Mueller Turnover Model [9] provide a rich theoretical perspective for researchers to explain the influencing mechanism of working conditions on turnover intention, and they are both suitable for the medical social work field. Unfortunately, most of them are limited to one single theoretical perspective—the Job Demands-Resources Model with job burnout as a mediating variable while the Price-Mueller Turnover Model with job satisfaction as a mediating variable, ignoring the correlation between job burnout and job satisfaction, resulting in the fragmentation and separation of the findings on the relationship between working conditions and turnover intention [10, 11]. Our study attempts to integrate the two theoretical models to synthetically analyze the multiple influencing mechanism of working conditions on the medical social workers’ turnover intention in the context of China to put forward suggestions for improving the medical social work conditions and expanding the medical social work professionals in China.

### Working conditions & turnover intention

The working conditions, as the most important exogenous influencing factors of employees’ turnover intention, refer to the preparation, creation, and maintenance of the environment for work task completion in organizations [12]. The limited changes in the working conditions over years and the continuous impacts of adverse working conditions on employees’ physical and psychological health have been found in recent years [13]. Employees will definitely leave their jobs unless their employers improve the poor working conditions [14]. The working conditions are composed of the external conditions and the internal conditions. Compared to the external ones (mainly including the political, economic, social, technical environment outside organizations), the internal ones, consist of the rules, regulations, as well as cultural atmosphere, are more controllable [12]. Therefore, the managers in organizations can improve the internal working conditions to enhance their management skills, and the organizations will further maintain stable human resources and achieve their goals and tasks efficiently. Turnover is a crucial part of organizational human resource management. It refers to the employees’ leaving rather than entering the organization [15], and the voluntary turnover behavior can directly reflect the internal management problems of the organization, so the managers should pay more attention to it [16]. Thus, the analysis of the formation mechanism of turnover intention is of great significance for improving organizations’ human resource management and promoting the healthy career development of employees. The existing theoretical perspectives for research on the working conditions at the organizational level and employees’ turnover intention mainly include the Job Demands-Resources Model with job burnout as a mediating variable and the Price-Mueller Turnover Model with job satisfaction as a mediating variable [8–11, 17]. The core perspectives of these two models and the influence relationships among these factors are as below.

### Working conditions, burnout, and turnover intention

Burnout is a significant predictor of turnover intention. Freudenberger, an American psychologist, was the first to introduce burnout into psychological research and defined burnout as the emotional failure or exhaustion of employees due to excessive demands on energy, physical strength, or resources [18]. Subsequently, Maslach and Jackson redefined burnout as the emotional and interpersonal stress responding to the high-load work on workers in the social services (human services) field, developed the first burnout inventory, Maslach Burnout Inventory-Human Services Survey (MBI-HSS), which consists of three dimensions: exhaustion, depersonalization, and reduced personal accomplishment [19]. The MBI-HSS is applicable to high emotionally demanding occupations such as medical social workers, and can effectively predict the formation of employees’ turnover intention in this type of occupation [20].

Working conditions are important influencing factors of job burnout [21]. Working conditions mainly include job demands and job resources [22]. In the Job Demands-Resources Model [17], job demands refer to the environmental, social, or organizational aspects of the job that require employees’ continuous physical and psychological efforts, resulting in the employees showing alienation from work tasks or service targets [8, 17]. Among all the job demands, job-related stress is the most important predictor of burnout [23]. Job-related stress mainly refers to the organizational risk factors including lack of sufficient resources, lack of clear rules and regulations, and unnecessary missions during completing tasks, leading the employees to pay associated physical, emotional, and cognitive fatigue and pressure [24]. Job resources are the supportive factors related to the environmental, social, and organizational aspects, including job autonomy and social support, which are functional in achieving work targets, reducing job demands at the associated physiological and psychological costs, and promoting employees’ growth and development [8, 17]. The Job Demands-Resources Model shows the influencing mechanism of the advantages and disadvantages in working conditions on employee burnout [8, 13, 25]. That is, job-related stress can lead to employee burnout [8, 11, 17, 25]. Various stressors and constraints will make employees more prone to emotional exhaustion and depersonalization, and social supports have a potential role in buffering employee burnout [8, 25]. Himle et al.’s research found that for social workers, the information support and tool support provided by colleagues and supervisors could effectively buffer job burnout caused by job-related stress [26].

Based on the above analysis, we can draw a critical path for how the working conditions affect turnover intention: the working conditions (including job demands and resources) will cause employee burnout, which will further aggravate their turnover intention [25, 27, 28]. Job burnout is a significant mediator between working conditions and employee turnover intention [8, 23].

### Working conditions, job satisfaction, and turnover intention

March and Simon first proposed a turnover model [29]. Since then, many scholars have also dissected the intermediate process of turnover intentions and behaviors [9, 30, 31], of which the most representative was the Price-Mueller Turnover Model. This model regards employees’ job satisfaction as a significant mediator connecting exogenous variables (such as working conditions) and voluntary turnover [9, 16] and believe that job-related stress, social support, and job autonomy as structurally environmental variables affect employees’ turnover intention through their job satisfaction. On the one hand, high job-related stress will affect employees’ job satisfaction through four stressful ways: inadequate resources (lack of means to perform a job), role ambiguity (unclear job obligations), role conflicts (inconsistent job obligations), and overload (excessive workload) [15]. They believe that these four ways make employees less satisfied with their jobs, further leading to a stronger turnover intention [32, 33]. On the other hand, high social support and job autonomy can improve employees’ job satisfaction. Social support can be categorized into three types: supervisor support, peer colleague support, and family support, of which supervisor and peer colleague support can effectively improve employees’ job satisfaction and reduce their turnover intention [27, 34, 35]. A study from Denmark showed that an unsupportive boss would increase the turnover rate by 6% [14]. Job autonomy is related to the size of the discretion the job has. Higher autonomy indicates more discretion and can make employees more satisfied with their jobs, resulting in lower turnover intention [36, 37].

The Price-Mueller Turnover Model explored the same influencing path with Gaertner [38], namely, job-related stress, social support, and job autonomy will affect job satisfaction and further influence turnover intention. This path has become the second critical one for the working conditions to affect their turnover intention.

### The connection between burnout and satisfaction

Scholars often use job burnout and job satisfaction as two independent variables in different theoretical models, discuss their formation mechanism and effects from different perspectives independently, seldom dissect the relationship between them [8, 9, 16, 17]. As mentioned above, job burnout is a crucial mediating variable between job demands and employees’ attitudes. In addition to turnover intention, another important attitude outcome of employees is job satisfaction [39]. According to Kaker’s research on the correlation between job satisfaction and burnout syndrome among health care social workers in Slovenia, it concluded that emotional exhaustion and depersonalization in job burnout were the key factors affecting social workers’ satisfaction with job characteristics and working conditions [40]. So it was necessary to monitor the job burnout of health care social workers and its potential impact on job satisfaction. It was helpful for social workers to maintain occupational health through psychological health [41]. Some other researchers had different views and believed that high job satisfaction was closely related to low burnout syndrome. They found that higher job satisfaction could reduce job burnout and further affected turnover intention [20, 42]. These two opposite opinions provide a novel explanation mechanism for in-depth exploration of the relationship between job burnout and job satisfaction, that is, when we pay attention to the influencing path of working conditions on turnover intention, we cannot ignore the correlation between job burnout and job satisfaction [43]. We need to conduct a more precise analysis process to discover a more detailed influencing mechanism in these factors.

The focus of each theoretical model varies. The job burnout model emphasizes the influence of physical fatigue and mental stress on turnover intention [21], while the job satisfaction model pays more attention to job recognition on turnover intention [35]. Any theoretical model is not independent; they show multiple influencing paths of working conditions on turnover intention, as well as new connections. Job demands and job resources will further affect employees’ attitude outcomes (including job satisfaction and turnover intention) through job burnout [11]. Therefore, when constructing the theoretical model, our study is more inclined that job burnout affects job satisfaction and test the relationship between the two with an empirical survey, to explore a more precise influencing mechanism of the working conditions on the turnover intention.

**Fig. 1:**
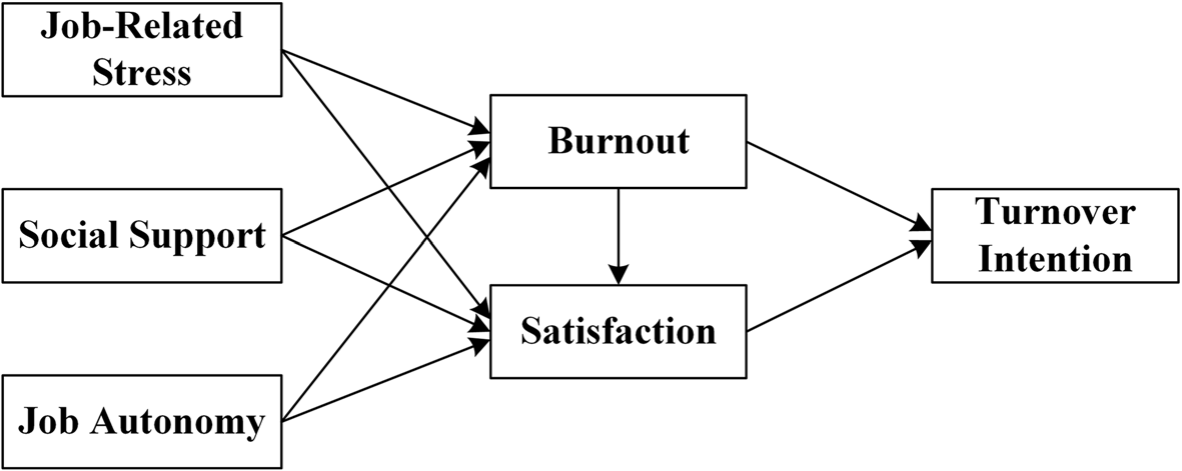
The theoretical framework

Based on the above review and analysis, we hypothesized that: (1_a_) the more job-related stress imposed on medical social workers, the higher their job burnout will be; (1_b_) the more social support the medical social workers obtain, the lower their job burnout will be; (1_c_) the more job autonomy the medical social workers have, the lower their job burnout will be; (1_d_) the higher the degree of medical social workers’ job burnout, the stronger their turnover intention will be; (1) job burnout plays a significant mediating role between working conditions and medical social workers’ turnover intention. (2_a_) the more job-related stress imposed on medical social workers, the lower their job satisfaction will be; (2_b_) the more social support the medical social workers obtain, the higher their job satisfaction will be; (2_c_) the more job autonomy the medical social workers have, the higher their job satisfaction will be; (2_d_) the higher the degree of medical social workers’ job satisfaction, the weaker their turnover intention will be; (2) job satisfaction plays a significant mediating role between working conditions and medical social workers’ turnover intention. (3_a_) the more job burnout the medical social workers have, the lower their satisfaction with their jobs will be; (3) adverse working conditions can increase job burnout of medical social workers, further reducing their job satisfaction and ultimately increasing the turnover intention. Figure 1 shows the theoretical framework of our study.

## Methods

### Participants and setting

Our study was conducted in the context of China. The data we used came from the China Social Work Longitudinal Survey (CSWLS) conducted in 56 cities across the country in 2019, the first nationwide investigation of social work agencies and social workers in China [4]. It adopted a multi-stage random sampling method and finally obtained 979 valid questionnaires for social work agencies and 6776 for social workers in the first phase. The sample of medical social workers was selected according to their current service field, by the question of “Which is your main service field now?”, and the sample size finally entering the model was 382.

### Measures

#### Dependent variable

The turnover intention was measured by three statements on a five-level Likert scale (1 = “strongly disagree” to 5 = “strongly agree”) [4]: “I plan to leave my current social work agency within the next six months”; “I may leave my current social work agency within the next three years”; and “Occasionally I have thoughts of leaving my current social work agency”. (KMO measure = 0.703, *p* < 0.001, Cronbach’s *α* = 0.830).

#### Mediating variables

Burnout was measured by the items adopted from Maslach Burnout Inventory-Human Services Survey (MBI-HSS), 22 items to measure three subscales consisting of emotional exhaustion, depersonalization, and reduced personal accomplishment [19]. Emotional exhaustion was measured through nine items: “I feel emotionally drained from my work”, “I feel used up at the end of the workday”, “working with people directly puts too much stress on me”, “working with people all day is really a strain for me”, “I feel burned out from my work”, “I feel I’m working too hard on my job”, “I feel frustrated by my job”, “I feel like I’m at the end of my rope”, “I feel fatigued when I get up in the morning and have to face another day on the job”. Depersonalization was measured through five items: “I’ve become more callous toward people since I took this job”, “I’ve become more callous toward people since I took this job”, “I don’t really care what happens to some recipients”, “I feel I treat some recipients as if they were impersonal ‘objects’, “I feel recipients blame me for some of their problems”. Personal accomplishment was measured through eight items: “I feel very energetic”, “I can easily understand how my recipients feel about things”, “I can easily create a relaxed atmosphere with my recipients”, “I feel exhilarated after working closely with my recipients”, “I have accomplished many worthwhile things in this job”, “I deal very effectively with the problems of my recipients”, “In my work, I deal with emotional problems very calmly”, “I feel I’m positively influencing other people’s lives through my work”. The respondents answered their personal feelings or attitudes stated in the items by the frequency they experienced, on a seven-level of ordinal variables (0 = “never” to 6 = “every day”). We calculated the mean values of each subscale as the final measure of the three components. (KMO measure = 0.548, *p* < 0.001, Cronbach’s *α* = 0.532).

Job satisfaction was measured by five items on a five-level Likert scale (1 = “strongly disagree” to 5 = “strongly agree”) [4]. The items were “I feel real joy in my job”; “I have an extraordinary job”; “I love my job more than other people”; “Most of the time I am passionate about my job”; and “I’m quite satisfied with my job”. (KMO measure = 0.849, *p* < 0.001, Cronbach’s *α* = 0.835).

#### Independent variables

Job demands and job resources are independent variables that were both measured by the five-level Likert scale (1 = “strongly disagree” to 5 = “strongly agree”).

In job demands, we mainly analyzed the construct of job-related stress. Job-related stress was measured by the items based on the Price-Mueller Turnover Model. Respondents answered the following items [9]: “I do some unnecessary tasks during the working time”; “My social work agency lacks corresponding rules and regulations to help me complete my tasks”; “The rules and regulations of my social work agency conflict with each other”; and “I lack sufficient resources to complete my tasks”. (KMO measure = 0.767, *p* < 0.001, Cronbach’s *α* = 0.802).

Job resources included social support and job autonomy. Social support consisted of four dimensions [44]: supervisor support, department manager support, peer colleague support, and top leadership support. Each dimension was measured by six items: “They are willing to provide reliable support when I encounter difficulties during work”; “They are willing to listen to the problems in my work”; “They are willing to help me complete the task together”; “They have the ability to do the job”; “They can handle their work tasks well”; “They will praise me for my outstanding performance”. The mean score of the six items of each subscale was calculated as the final measure for the four dimensions. (KMO measure = 0.806, *p* < 0.001, Cronbach’s *α* = 0.846). Job autonomy was measured by three items [45]: “I have the discretion to decide whether or not to undertake a task”; “I can decide how to conduct my work”; and “My opinions are influential in the work discussion”. (KMO measure = 0.710, *p* < 0.001, Cronbach’s *α* = 0.812).

#### Control variables

We regarded the respondents’ demographics, including gender, age, marital status, monthly income, education years, and years of engaging in medical social work as control variables. Table 1 shows the results of the descriptive statistics of all variables.

**Table 1:** Results of the descriptive statistics of variables (*n* = 382)

Latent Variables	Code	Measurement	Mean	S.D.
Turnover Intention	Y1	I plan to leave my current social work agency within the next six months (1–5)	1.948	1.026
	Y2	I may leave my current social work agency within the next three years (1–5)	2.484	1.108
	Y3	Occasionally I have thoughts of leaving my current social work agency (1–5)	2.652	1.215
Job Burnout	Y4	Emotional exhaustion (0–6)	1.339	1.096
	Y5	Depersonalization (0–6)	.425	.781
	Y6	Personal accomplishment (0–6)	4.161	1.399
Job Satisfaction	Y7	I feel real joy in my job (1–5)	3.767	.753
	Y8	I have an extraordinary job (1–5)	3.641	.793
	Y9	I love my job more than other people (1–5)	3.647	.779
	Y10	Most of the time I am passionate about my job (1–5)	3.395	.690
	Y11	I’m quite satisfied with my job (1–5)	3.602	.796
Job-related stress	X1	I lack sufficient resources to complete my tasks (1–5)	2.950	.975
	X2	I do some unnecessary tasks during the working time (1–5)	2.798	1.021
	X3	My social work agency lacks corresponding rules and regulations to help me complete my tasks (1–5)	2.791	.987
	X4	The rules and regulations of my social work agency conflict with each other (1–5)	2.702	.967
Social Support	X5	Supervisor support (1–5)	3.901	.979
	X6	Department manager support (1–5)	4.024	.967
	X7	Peer colleague support (1–5)	4.109	.836
	X8	Top leadership support (1–5)	3.815	1.071
Job Autonomy	X9	I have the discretion to decide whether or not to undertake a task (1–5)	3.419	.853
	X10	I can decide how to conduct my work (1–5)	3.518	.815
	X11	My opinions are influential in the work discussion (1–5)	3.518	.769
Control Variables	X12	Gender(1 = Male, 0 = Female)	.275	.447
	X13	Age	31.749	7.971
	X14	Marital status(1 = Married, 0 = Unmarried)	.531	.500
	X15	Monthly income(RMB, ten thousand)	.428	.242
	X16	Education years (year)	16.275	2.173
	X17	Length of time working in medical social work (year)	4.42	3.640

### Statistical analysis

**Fig. 2:**
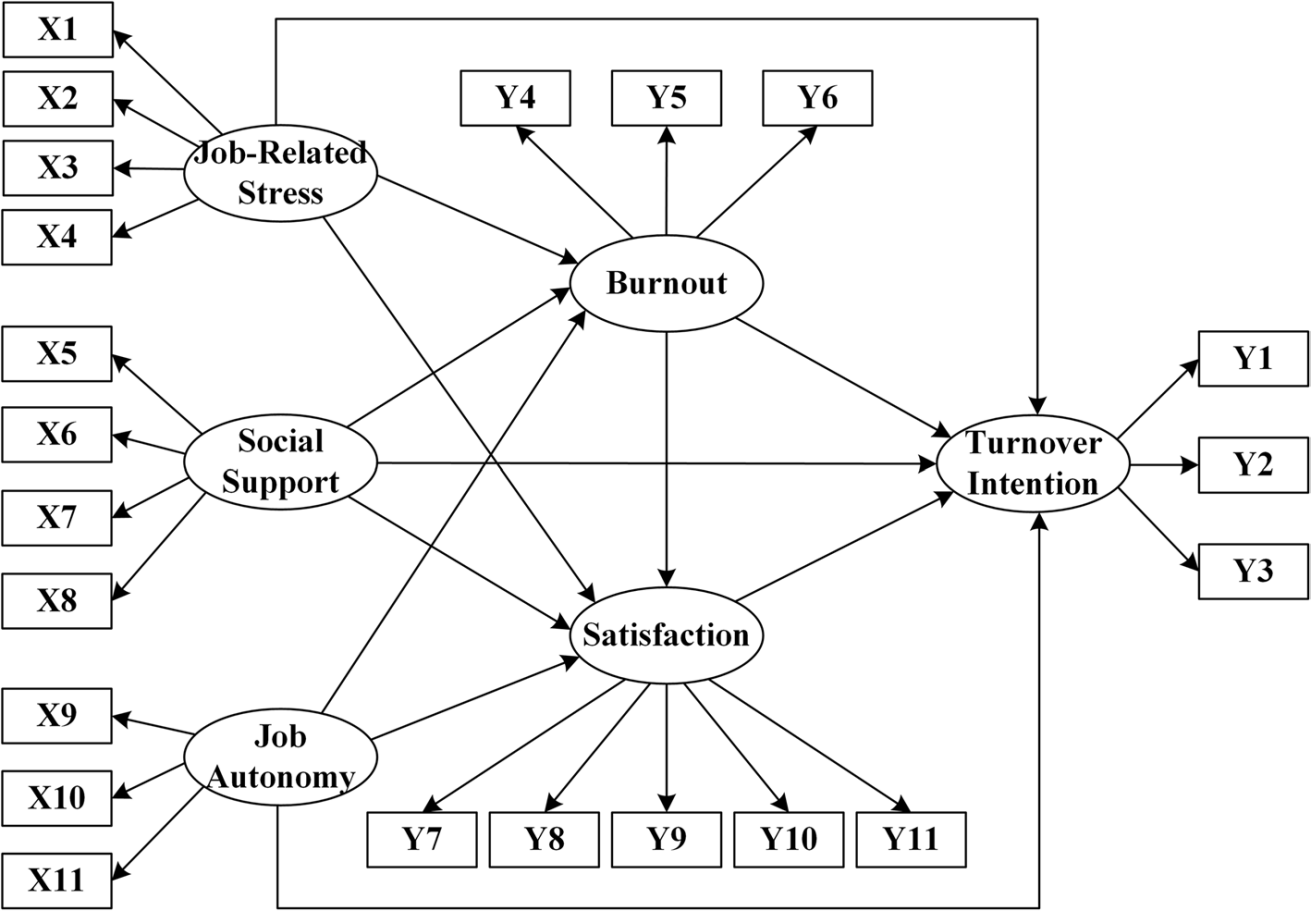
The structural equation model of this study

We tested the relationships between working conditions and turnover intention with the structural equation model (SEM) by STATA 16.0. The SEM can estimate both the relationships between observed variables (i.e., measurement indicators) and latent variables (or constructs) as well as the relationships between latent variables. A structural equation model (see Fig. 2) was set based on the theoretical framework (see Fig. 1) and the hypotheses above. The relationships between the latent variables and correspondence observed variables (see Table 1) were as follows: Firstly, job-related stress, social support, and job autonomy can impact the turnover intention of medical social workers indirectly through job burnout and job satisfaction. Thus, in our study, we set job-related stress, social support, and job autonomy as exogenous latent variables. The existing studies have shown that the exogenous variables can significantly influence both job burnout and job satisfaction. Secondly, job burnout can not only directly impact the turnover intention of medical social workers, but also can indirectly impact it through job satisfaction. Thirdly, there were no direct measurement indicators for social support and job burnout. In our model, they were estimated as the mean of the latent variables of various dimensions. For the model’s simplicity, the control variables were not shown in the structural equation model, but they were controlled in the data analysis.

## Results

Evaluating the fitting indices of the structural equation model is the prerequisite for explaining the relationship between the measurement model and the latent variables. In general, these indices include Chi-square (χ^2^), Chi-square/df (χ^2^/df), the Root Mean Squared Error of Approximation (RMSEA), Standardized Root Mean Square Residual (SRMR), the Goodness of Fit Index (GFI), the Adjusted Goodness of Fit Index (AGFI), the Comparative Fit Index (CFI), and the Tucker-Lewis Index (TLI) [46, 47]. χ^2^ is the most commonly reported goodness of fit index, and when used with degrees of freedom, χ^2^/df can directly test the similarity between sample covariance matrix and estimate variance matrix, and explain the probability of model correctness. Its theoretical expected value is 1, and in the actual research, χ^2^/df close to 2 indicates that the fitting of the model is good [48]. As shown in Table 2, for our structural model, the χ^2^/df was 2.161, close to 2, the values for RMSEA (0.055) and SRMR (0.056) was less than 0.080, and the values for GFI (0.910), AGFI (0.920), CFI (0.905) and TLI (0.881) were all close to 1.000, indicating a good model fitting. Table 2 shows the fitting indices of the structural model.

**Table 2:** Model fit indices of the structural model

Indices	Estimates
χ^2^	626.607
df	290
χ^2^/df	2.161
RMSEA	0.055
SRMR	0.056
GFI	0.910
AGFI	0.920
CFI	0.905
TLI	0.881
N	382

Table 3 summarizes the standardized loadings of the observed variables to the latent variables. The measurement model demonstrated a high convergent validity, as the standardized factor loadings of all observed variables (except personal accomplishment) were higher than 0.5; all *p* values for their loadings were significant. We could find that the relatively low standardized factor loading of personal accomplishment (*β* = − 0.311, *p* < 0.001) indicates that personal accomplishment could not fully reflect the difference of job burnout of medical social workers in our study. One explanation could be that the MBI-HSS scale may not be fully applicable to the Chinese context. We expect that further research can make up for the shortcomings in measuring job burnout in the Chinese context.

**Table 3:** Properties of the structural model (*N* = 382)

Construct	Indicator	SFL	SMC	Construct	Indicator	SFL	SMC
Turnover Intention	Y1	.719^***^	.516	Job-Related Stress	X1	.628^***^	.394
	Y2	.870^**^	.757		X2	.786^***^	.618
Job Burnout	Y3	.787^***^	.619	Social Support	X3	.774 ^***^	.599
	Y4	.780^***^	.608		X4	.655^***^	.428
	Y5	.750^***^	.562		X5	.759^***^	.576
Job Sanctification	Y6	−.311^***^	.097	Job Autonomy	X6	.876^***^	.767
	Y7	.819^***^	.671		X7	.754^***^	.569
	Y8	.729^***^	.531		X8	.688^***^	.473
	Y9	.805^***^	.649		X9	.817^***^	.668
	Y10	.431^***^	.186		X10	.715^***^	.511
	Y11	.756^***^	.572		X11	.775^**^	.601

Notes: The first indicator of a latent variable is the reference scale; ** *p* < 0.01; *** *p* < 0.001

Table 4 and Table 5 are the results of validity test. As shown in Table 4, except for burnout, the composite reliability (CR) of each construct was higher than 0.70 while the average variance extracted (AVE) of each construct was higher than 0.50, indicating that the items of the constructs had good convergent validity. We will modify the model later by trimming the low loading factors in terms of the poor validity of burnout.

**Table 4:** Convergent validity

Construct	Composite Reliability (CR)	Average Variance Extracted (AVE)
Job-related stress	0.804	0.510
Job Autonomy	0.814	0.593
Social Support	0.854	0.596
Job Burnout	0.661	0.423
Job Satisfaction	0.840	0.522
Turnover Intention	0.836	0.630

**Table 5:** Discriminative validity

	Job-related stress	Job Autonomy	Social Support	Job Burnout	Job Satisfaction	Turnover Intention
Job-related stress	0.713					
Job Autonomy	−0.162^**^	0.770				
Social Support	−0.334^***^	0.172^***^	0.772			
Job Burnout	0.341^***^	−0.186^***^	−0.134^**^	0.650		
Job Satisfaction	−0.284^***^	0.329^***^	0.191^***^	−0.379^***^	0.722	
Turnover Intention	0.288^***^	−0.038	−0.141^**^	0.215^***^	−0.290^***^	0.794

Notes: (1) The diagonal number is the square root of AVE. (2) Significance of two-tailed test: ** *p* < 0.01; *** *p* < 0.001

Table 5 shows the Pearson correlation results and the square root of the average variance extracted of each construct. The results indicated a good discriminative validity because the square root of the average variance extracted of the constructs were all greater than the correlation coefficient between the construct and other constructs.

**Fig. 3:**
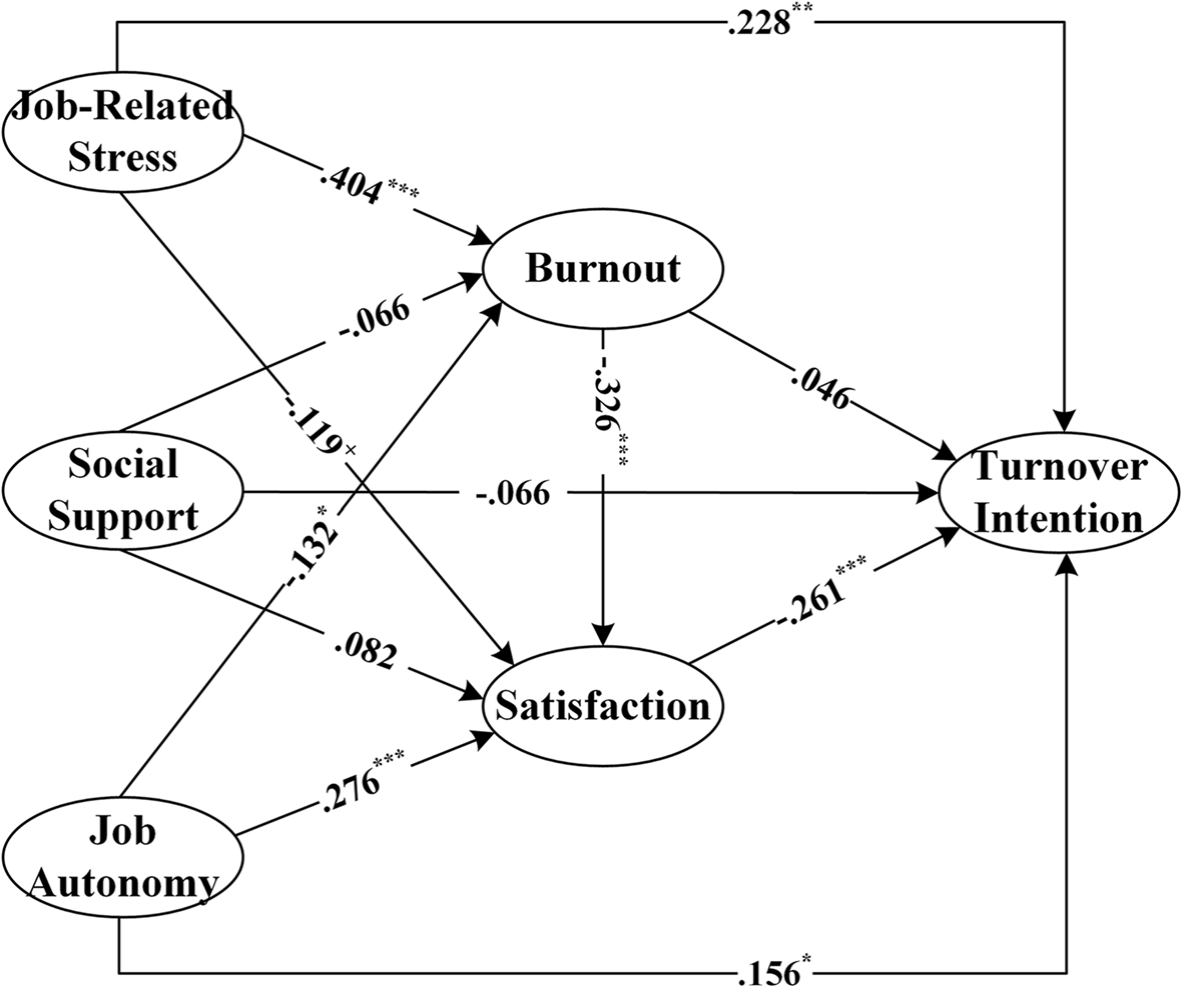
Path diagram of the working conditions affecting turnover intention of medical social workers

Path analysis of the impact of working conditions on turnover intention of medical social workers was conducted and the path diagram and the Standardized path coefficients were shown in Fig. 3 and Table 6.

**Table 6:** Standardized path coefficients (*N* = 382)

	Job Burnout	Job Satisfaction	Turnover Intention
Job-Related Stress	.404^***^ (.065)	−.119^+^ (.067)	.228^**^ (.068)
Social Support	−.066 (.070)	.082 (.060)	−.066 (.062)
Job Autonomy	−.132^*^ (.066)	.276^***^ (.056)	.156^*^ (.063)
Job Burnout		−.326^***^ (.064)	.046 (.019)
Job Satisfaction			−.261^***^ (.069)

Notes: Exogenous variables are listed in row; Endogenous variables are listed in column; Standard errors are marked in parentheses; + *p* < 0.1; * *p* < 0.05; ** *p* < 0.01; *** *p* < 0.001

Overall, the model explained 21.27% of the data variation in job burnout, 35.04% of the data variation in job satisfaction, and 28.43% of the data variation in turnover intention.

### Hypothesis 1

The quality of the working conditions significantly determines the level of job burnout of medical social workers. Figure 3 and Table 4 shows that the standardized path coefficient of job-related stress on job burnout was 0.404 (*p* < 0.001), if job-related stress increases by each additional standard unit, and job burnout of medical social workers will increase by 0.404 standard units, which supported Hypothesis 1_a_. The standardized path coefficient of the influence of social support on job burnout was − 0.066 (*p* > 0.1), which was not significant, indicating that social support had no significant impact on job burnout. Hypothesis 1_b_ was not supported by data. The standardized path coefficient of the influence of job autonomy on job burnout was − 0.132 (*p* < 0.05), which was significant, indicating that job autonomy can significantly reduce job burnout. Hypothesis 1_c_ was supported. The coefficient of the path from job burnout to turnover intention was 0.046 (*p* > 0.1), not significant, indicating that job burnout did not have a direct impact on the turnover intention of medical social workers. Thus, Hypothesis 1_d_ was not supported. Based on the above results, H_1_ was not supported.

### Hypothesis 2

Firstly, job-related stress had a significant negative impact on the job satisfaction of medical social workers (*β* = − 0.119, *p* < 0.1). The job satisfaction of medical social workers would decrease by 0.119 standard units if the job-related stress increased by each additional standard unit. Thus, hypothesis 2_a_ was supported by the data. Secondly, job autonomy could significantly improve the job satisfaction of medical social workers (*β* = 0.276, *p* < 0.001). The satisfaction of medical social workers would increase by 0.276 standard units if the job autonomy increased by each additional standard unit. Therefore, Hypothesis 2_c_ was also supported. However, the effect of social support on job satisfaction was not significant, so Hypothesis 2_b_ had not been verified. The impact of job satisfaction on turnover intention was − 0.261 (*p* < 0.001), indicating that the job satisfaction of medical social workers significantly reduced turnover intention, which supported Hypothesis 2_d_. Based on the above results, H_2_ was supported.

### Hypothesis 3

The impact of job burnout on job satisfaction was − 0.326 (*p* < 0.001), indicating that job burnout had a significant negative impact on job satisfaction, which supported Hypothesis 3_a_. Based on the above results, we can conclude that job satisfaction had a fully mediating effect between job burnout and turnover intention of medical social workers. That is, job burnout did not have a direct impact on the turnover intention of medical social workers; instead, it impacted turnover intention of medical social workers indirectly through job satisfaction. Based on the results above, H_3_ was supported.

### The total effects of working conditions on turnover intention

Table 7 reports the standardized total effects of each working conditions factor on turnover intention. As shown in Table 5, job-related stress had the most significant total effect on turnover intention (*β* = 0.307, *p* < 0.001), followed by job satisfaction (*β* = − 0.261, *p* < 0.001) and job burnout (*β* = 0.131, *p* < 0.1). However, the total effects of social support (*β* = − 0.078, *p* > 0.1) and job autonomy (*β* = 0.066, *p* > 0.1) on turnover intention were not significant. These results indicated that the turnover intention of medical social workers was largely influenced by the job-related stress of the social work agency. Further, the impact of job-related stress on turnover intention was not simple and direct. Job-related stress could increase the job burnout and decrease their job satisfaction of medical social workers, which would, in turn, lead to increased turnover intention.

**Table 7:** The standardized total effects of working conditions on turnover intention (*N* = 382)

	Job Burnout	Job Satisfaction	Turnover Intention
Job-Related Stress	.404^***^ (.065)	−.251^***^ (.064)	.307^***^ (.084)
Social Support	−.066 (.070)	.060 (.051)	−.078 (.063)
Work Autonomy	−.132^*^ (.066)	.319^***^ (.054)	.066 (.064)
Job Burnout		−.326^***^ (.052)	.131^+^ (.061)
Job Satisfaction			−.261^***^ (.087)

Notes: Exogenous variables are listed in row; Endogenous variables are listed in column; Standard errors are marked in parentheses; + *p* < 0.1; * *p* < 0.05; *** *p* < 0.001

### The modified model

**Fig. 4:**
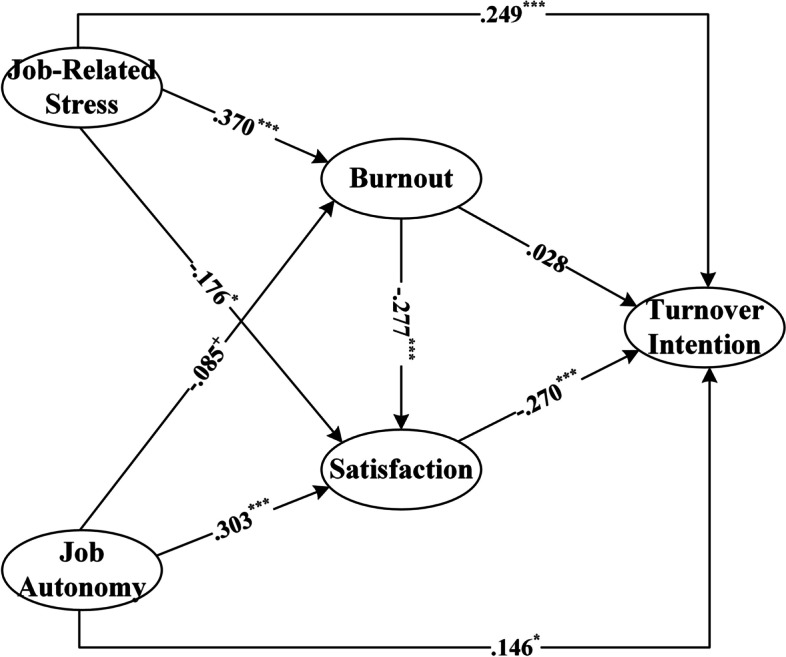
The modified model

We modified the model by trimming the low loading factors and insignificant hypotheses and paths. The results showed that the modified model had a better model fitting (χ^2^/df = 2.034, RMSEA = 0.052, SRMR = 0.046, GFI = 0.922, AGFI = 0.921, CFI = 0.929, TLI = 0.907) and a better validity (CR: Job-related stress = 0.804, Job Autonomy = 0.814, Job Burnout = 0.762, Job Satisfaction = 0.840, Turnover Intention = 0.836; AVE: Job-related stress = 0.509, Job Autonomy = 0.593, Job Burnout = 0.619, Job Satisfaction = 0.522, Turnover Intention = 0.630; the square root of AVE: Job-related stress = 0.713, Job Autonomy = 0.770, Job Burnout = 0.787, Job Satisfaction = 0.722, Turnover Intention = 0.794). Figure 4 shows the final model.

## Discussion

The development of medical social work is an indispensable part of the Healthy China Strategy, and the retention of professionals is a necessary guarantee for the long-term development of medical social work. Taking medical social work in China as an example, our study analyzed the first-phase data from the China Social Work Longitudinal Survey (CSWLS) in 2019 and tried to explore the influencing mechanism of the working conditions on the turnover intention of medical social workers. Eventually, we draw the main conclusion: the environmental factors at the organization level are the crucial antecedent variables to ensure social workers staying in the field of medical services. Among them, job-related stress plays the most significant role in explaining the formation mechanism of medical social workers’ turnover intention [49]. On the one hand, job-related stress can reduce the job satisfaction of medical social workers, further increasing their turnover intention, which verified the core perspective of Price-Mueller turnover model [9, 16]; on the other hand, job-related stress can increase job burnout of medical social workers, further reducing their job satisfaction and ultimately increasing the turnover intention. Job satisfaction plays a full mediating effect between the job burnout of medical social workers and their turnover intention. It was worth noting that the job resources provided by social work agencies had limited effects on decreasing the turnover intention of medical social workers, and the total effects of social support and job autonomy on turnover intention were small and not significant, all of which suggested that having more social support and work autonomy could not prevent medical social workers from having a turnover intention. This finding is different from Kim and Stoner’s study. They analyzed the influence mechanism of job demands and job resources on social workers’ turnover intention with job burnout as the mediating variable, and found that job resources including social support and job autonomy, would have direct negative effects on social workers’ turnover intention [5]. This indicates that the impact of job resources on turnover intentions in the field of medical social work presents unique characteristics compared to the overall social work fields. Nevertheless, higher job autonomy can still reduce the job burnout of medical social workers and increase their job satisfaction significantly, which is consistent with the conclusion by Yanchus et al. who found that autonomy predicted job satisfaction [32], and the conclusion by Aronsson et al. who discovered the significant impact of low job control on burnout symptom [22]. Therefore, the medical system should give medical social workers more job autonomy and discretion to guarantee their lower job burnout and higher job satisfaction.

## Conclusions

### Theoretical implications

Creating favorable working conditions can not only reduce the instability of social workers in the medical service field but also can attract more social workers to engage in this field. This study discovers the full mediating effect of job satisfaction and two influencing paths between the job burnout of medical social workers and their turnover intention. The two paths of job-related stress affecting turnover intention successfully integrate the Job Demands-Resources Model and the Price-Mueller Turnover Model into the comprehensive theoretical framework, complementing the existing literature’s lack of research on the relationship between working conditions at the organizational level and individual turnover intention in medical social work. Otherwise, it also provides a theoretical basis for reducing the turnover intention and behavior of social workers in the medical service field, improving the management level in the medical service system, and promoting the overall healthy and sustainable development of medical social work.

### Practical implications

Our research suggestions are as below: First of all, managers in the medical service system should pay more attention to the unfavorable factors in the working conditions of medical social workers, help them set their daily working goals, choose the correct working methods, and give medical social workers sufficient guidance. Those approaches can reduce the high job burnout and low job satisfaction caused by job-related stress [5]. Secondly, in particular, preventive monitoring should be carried out on the dominant elements of crucial mediating variables, such as timely detection of dissatisfaction and complaints expressed by medical social workers and creating a supportive working environment [35]. Preventive monitoring can significantly improve the internal management level and reduce the unnecessary human resource management costs caused by frequent turnover in the medical services system. Finally, at the policy level, unclear policies and corresponding management strategies may impose many incompatible demands on medical social workers [50], increasing their possibility of turnover. Therefore, governments and departments at all levels should optimize relevant policies based on the characteristics of medical social work development in China, support the construction of professionals, provide a favorable organizational environment, and further protect medical social workers staying in the medical services field.

### Limitations and suggestions for future research

First, we only used cross-sectional data in this paper, so we couldn’t figure out the causal relationship of the factors. Second, the standardized factor loading of personal accomplishment on burnout was relatively low indicating that personal accomplishment could not fully reflect the difference of job burnout of medical social workers in our study. Third, we only tested several critical factors of job demands and job resources, resulting in the limited explanatory power of the research conclusions. Future research worthy of in-depth analysis: firstly, supplement the second-phase data of CSWLS to explore the causal mechanism between the working conditions and turnover intention of medical social workers; secondly, the MBI-HSS scale’s measurement of job burnout needs to be revised based on Chinese settings; thirdly, more factors in job demands and job resources, and their interactive effects need to be further tested.

## Data Availability

The datasets used and/or analyzed during the current study are available from the corresponding author on reasonable request.

## Abbreviations

SD: Standard Deviation
SEM: Structural Equation Model
RMSEA: Root Mean Square Error of Approximation
CFI: Comparative Fit Index
TLI: Tucker-Lewis Index
SFL: Standardized Factor Loading
SMC: Squared Multiple Correlation
